# Expanding the catalog of *cas* genes with metagenomes

**DOI:** 10.1093/nar/gkt1262

**Published:** 2013-12-05

**Authors:** Quan Zhang, Thomas G. Doak, Yuzhen Ye

**Affiliations:** ^1^School of Informatics and Computing, Indiana University, Bloomington, IN 47405, USA, ^2^Department of Biology, Indiana University, Bloomington, IN 47405, USA and ^3^National Center for Genome Analysis Support, Indiana University, Bloomington, IN 47408, USA

## Abstract

The CRISPR (clusters of regularly interspaced short palindromic repeats)–Cas adaptive immune system is an important defense system in bacteria, providing targeted defense against invasions of foreign nucleic acids. CRISPR–Cas systems consist of CRISPR loci and *cas* (CRISPR-associated) genes: sequence segments of invaders are incorporated into host genomes at CRISPR loci to generate specificity, while adjacent *cas* genes encode proteins that mediate the defense process. We pursued an integrated approach to identifying putative *cas* genes from genomes and metagenomes, combining similarity searches with genomic neighborhood analysis. Application of our approach to bacterial genomes and human microbiome datasets allowed us to significantly expand the collection of *cas* genes: the sequence space of the Cas9 family, the key player in the recently engineered RNA-guided platforms for genome editing in eukaryotes, is expanded by at least two-fold with metagenomic datasets. We found genes in *cas* loci encoding other functions, for example, toxins and antitoxins, confirming the recently discovered potential of coupling between adaptive immunity and the dormancy/suicide systems. We further identified 24 novel Cas families; one novel family contains 20 proteins, all identified from the human microbiome datasets, illustrating the importance of metagenomics projects in expanding the diversity of *cas* genes.

## INTRODUCTION

Clustered regularly interspaced short palindromic repeats (CRISPR) associated (Cas) protein systems are RNA-guided adaptive immunity system that provides bacteria with sequence-directed defense against invading DNAs or RNAs (1–7). CRISPR–Cas systems are present in a majority of archaeal genomes and in many bacterial genomes (3,8–10). In general, CRISPR spacer-repeat arrays consist of 24- to 47-bp direct repeats flanking unique spacers acquired from foreign DNAs that have invaded the host and been stored in CRISPR arrays as a consequence. To affect interference, these arrays are transcribed as precursor RNAs, and subsequently truncated to short CRISPR RNAs by Cas proteins, and then used to guide attacks on the matching protospacers in invading genomes, using other Cas activities (8,9). CRISPR arrays consist of from several to a few hundred repeat-spacer units (11).

It is generally understood that there are three stages in the silencing of foreign nucleic acids by the CRISPR–Cas systems: adaptation, expression and interference (12). Cas proteins—proteins encoded by the *cas* genes located in the genomic neighborhood of the CRISPR arrays—are found to play important roles in each of these stages (6). The adaptation stage is also referred to as the ‘information processing’ subsystem, and is likely to involve highly conserved proteins, including Cas1 and Cas2. The later two stages (expression and interference) are referred to as the ‘executive’ subsystem, and involve proteins that are highly variable among different *cas* loci (12).

The first widely used Cas protein classification system—based on 200 complete prokaryotic genomes—included 45 Cas protein families divided into eight subtypes (13). Each subtype is found in the genome that it was named after (13). A later study by Makarova *et al.* divided the major types of Cas protein families into more subtypes, based on 703 archaeal and bacterial genomes, using a phylogenetic classification (12). Based on the different participating Cas proteins, CRISPR–Cas immune systems are now divided into three main types (types I, II and III)—each can be further classified into subtypes (e.g. *Escherichia coli* has a sub-type IE CRISPR system).

Some core proteins are found to be universally present in all three types (1,14): Cas1 proteins are involved in the adaptive integrating of foreign nucleic acids into CRISPR arrays, as studied in *Pseudomonas aeruginosa* (15); Cas2 is a metal-dependent endoribonuclease, whose role in the CRISPR–Cas pathway remains unclear (16). The subtype I-E executive system is comprised of five Cas proteins (including Cas1 and Cas2), which have been experimentally demonstrated to form a nucleoprotein complex with CRISPR RNA (crRNA) for antiviral defense in *E. coli* K12 (14). On the other hand, a complex composed of Cas RAMP (repeat associated mysterious proteins) superfamily modules (Cmr) and crRNA is able to target invading RNA (17) (subtype III-B) or DNA (18) (subtype III-A) sequences in the type III CRISPR–Cas system. The type II CRISPR–Cas system from *Streptococcus pyogenes* is the simplest executive system, with a single gene encoding Cas9 protein and two RNAs: a mature CRISPR RNA (crRNA) and a partially complementary trans-acting RNA (tracrRNA) (the tracrRNA family has no obvious conservation of structure, sequence or localization within type II CRISPR-Cas loci (19)). The type II system is sufficient for RNA-guided silencing of foreign DNAs (20,21). Recently this system has been engineered to achieve guided genome engineering in human cells (21,22), *Saccharomyces cerevisiae* (23), and zebrafish embryos (24), and to achieve selective repression of gene expression in *E. coli* (by cleverly using a catalytically dead Cas9 lacking endonuclease activity) (25). However, the role of many types of Cas proteins, including Cas2 (16), in the defense process is still unclear.

Several studies have been conducted to better understand the CRISPR–Cas system in metagenomes collected from, for example, hot spring microbial mats (16), the *Sorcerer II* Global Ocean Sampling expedition (26), and the Human Microbiome Project (27), but have focused on the CRISPR arrays—few studies have analyzed Cas protein families using metagenomic data. In this article, we utilized the large collection of human microbiome project (HMP) datasets (28,29) to identify potential *cas* genes (and their proteins) in the HMP datasets. Our approach to identifying *cas* genes takes advantage of the existing classification of Cas proteins and the fact that *cas* genes are usually found clustered in genomes (the *cas* loci), adjacent to CRISPRs. Similarity searches using known Cas proteins are used to find seed *cas* regions in complete or draft bacterial genomes and in metagenome assemblies (contigs); the seed *cas* regions are then expanded to include putative *cas* genes in their genomic neighborhoods. We find genes in *cas* loci which encode other functions, for example, toxins and antitoxins, confirming the recently discovered potential of coupling adaptive immunity and dormancy/suicide systems in bacteria. Our study resulted in a large collection of *cas* genes (and their proteins) with many new families, and provided a more comprehensive view of the *cas* genes and their distributions in different microbial communities.

## MATERIALS AND METHODS

### Identification of cas genes from complete/draft genomes and metagenome assemblies

We obtained Cas protein family Hidden Markov Models (HMM) from the TIGRFAMs database (version 13.0) (30) (www.tigr.org/TIGRFAMs), since it is the common database used for studies of Cas proteins (12,13). We also included PFAM (31) families that are annotated as Cas families (downloaded from ftp://ftp.ncbi.nih.gov/pub/wolf/_suppl/CRISPRclass/crisprPro.html). In total, we collected 130 known Cas protein families: 99 TIGRFAMs and 31 PFAM Cas families; we used these known Cas families as the reference for the identification of Cas proteins based on similarity searches.

Complete and draft genomes, and their gene predictions, were downloaded from the NCBI ftp website (ftp.ncbi.nlm.nih.gov). We downloaded the whole metagenome assemblies and gene annotations for the HMP datasets from the DACC website (http://hmpdacc.org).

We first identify genes that encode putative Cas proteins based on similarity searches using known Cas protein families. We used hmmscan in the hmmer package (version 3.0) (32) (using the gathering cutoff) to assign proteins to known TIGRFAM families. These genes are used as seeds to recruit more putative *cas* genes that share only weak similarities with known Cas proteins (with *E*-value ≤ 0.001), in the genomic neighborhood of the seed genes (within a three-gene-distance on either side of any of the seed genes). We used three genes to define the neighborhood since most of the known *cas* loci have at least three genes (12). Recruited genes are used as seed genes for further recruitment; this recruitment of neighborhood genes is repeated until no more genes can be recruited. Thus, the final set of genes includes the seed known *cas* genes, nearby known *cas* genes of low similarity (recruited), and genes without known *cas*-gene affinities that fall between *cas* genes. For the recruited genes that encode proteins sharing no similarities with known Cas proteins, we searched their proteins against the entire TIGRFAM and PFAM databases to annotate their potential functions. PFAM database (version 27.0) was downloaded from the ftp site (ftp://ftp.sanger.ac.uk/pub/databases/Pfam/). We considered proteins that could not be annotated to any of the TIGRFAM and PFAM families as putative novel Cas proteins.

### Type/subtype classification

We assigned types/subtypes to predicted *cas* loci and therefore to the novel cas families found in these loci. A *cas* locus is assigned to a type (or subtype) if the corresponding signature gene is found in the locus. We used all three main types (types I, II and III) and 11 subtypes as defined in (12,13) [see Table 2 in the reference (12)], and subtype IC-variant from (33). We note that for type II, we considered all three subtypes II-A (with subtype signature gene *csn*2), II-B (with subtype signature gene *cas*4) and II-C (which only has *cas*9, *cas*1 and *cas*2 genes) (42) when assigning subtypes to the *cas* loci found in complete genomes; in contrast, we only considered II-A and II-B for the *cas* loci found in draft genomes or the HMP assemblies, as an incomplete *cas* locus may be incorrectly assigned to subtype II-C.

### Clustering of novel putative Cas proteins using Tribe-MCL

We first derived a set of redundant proteins, with 90% identity, using the CD-HIT program (34). We then performed an all-to-all BLASTP search of our non-redundant novel putative Cas proteins and selected pairs with BLAST bit scores greater than 60 (35). Subsequently, we applied Tribe-MCL (36) to cluster the putative novel Cas proteins into groups based on their sequence similarity. We used 1.4 as the inflation parameter, and default values for the other parameters for the clustering—we tried different inflations parameters, including the ones suggested by the Tribe-MCL website (http://micans.org/mcl/man/clmprotocols.html), and the clustering results were most reasonable (based on manual checking) when an inflation of 1.4. Cytoscape (37) was used to visualize the clustering results. We manually checked the genomic contexts of the *cas* genes in each cluster.

### Phylogenetic tree reconstruction

We utilized MUSCLE (38) for protein alignment, and MEGA (http://www.megasoftware.net/) to construct phylogenetic trees, using neighbor-joining and 500 bootstrap replications for the novel putative Cas families. For Cas9 proteins, the FastTree program (39) with default parameters was used to reconstruct the neighbor-joining tree.

### Building profile HMMs for novel putative Cas proteins

We built a profile HMM for each of the 24 families of novel putative Cas proteins. We excluded short sequences: those two standard deviations or more away from the mean of each family’s length distribution. The remaining sequences of each family were then aligned using MUSCLE (38) and used as the initial seed sets for HMM building using hmmbuild from the HMMER package. We then iteratively refined the HMM of each family by recruiting sequences from the bacterial database (40) that scored higher than the worst scoring seed sequence. For each iteration, a new HMM was built with the additional sequences added and the worst seed sequence removed. The iteration was terminated when no additional sequences were detected. For all 24 families, the HMM refinement procedure was completed within three iterations. The lowest score of the final seed set was recorded as the gathering and trusted cutoff, and the highest score of a sequence that was not in the seed set was recorded as the noise cutoff.

### Estimating Cas gene percentages in different body habitats

We selected 65 subjects who had *cas* genes in all stool and sub-oral habitats. For each sample, the *cas* gene percentage was calculated by the number of annotated *cas* genes, including the *cas* genes annotated by similarity searches and our newly discovered *cas* genes, divided by the total number of all genes collected from each body habitat. To compare the difference of the *cas* gene percentage between the stool and oral habitats, we utilized a two-tail paired *t*-test.

### Availability of the Cas proteins and their genes

Predicted *cas* genes and their proteins are available for download at our website http://omics.informatics.indiana.edu/mg/CAS. Other information, including the genomic contexts, phylogenetic trees and profile HMMs of all 24 novel Cas families are also available at the website.

## RESULTS

### Identification of putative cas genes

Our approach starts by collecting genes encoding proteins that share significant similarities to known Cas proteins, and then expands the collection by including genes within three genes of these *cas* genes that are already in the known *cas* collection, even though they may share only weak similarities with known Cas proteins. In total we identified 13 182 known *cas* genes (with significant similarities or weak similarities, recruited because they are close to the significant ones) from complete genomes, 18 318 from draft genomes, and 131 117 from the HMP assemblies (see Supplementary Tables S1 and S2 for a breakdown of the identified genes for selected Cas families, including core and type/subtype specific families). We also classified the *cas* loci we identified into different types/subtypes of CRISPR–Cas systems, based on the presence of type-specific Cas families (see Table 1 for details). Overall, the type-I CRISPR–Cas system is more prevalent than the other two types in human microbiomes, similar to the distribution inferred from reference genomes.

**Table 1.** Distributions of major types and subtypes of CRISPR-Cas systems in reference genomes and human microbiomes

Type	Number complete genomes	Number draft genomes	Number HMP contigs^a^	Subtype	Number complete genomes	Number draft genomes	Number HMP contigs
I	909	2051	10 497	I-A	165	240	849
				I-B	209	205	932
				I-C	214	418	4139
				IC-variant^b^	59	44	512
				I-D	56	30	5
				I-E	280	941	2851
				I-F	125	221	305
II	209	437	5244	II-A	71	117	524
				II-B	12	16	28
				II-C	127	-^c^	- ^c^
III	403	307	5207	III-A	249	149	3270
				III-B	190	105	576

^a^Contigs with at least two *cas* genes for this statistics are included.

^b^Subtype IC-variant is from the reference (33).

^c^The subtype II-C in draft genomes or HMP contigs did not quantified, considering that a *cas* loci may be falsely assigned to this subtype when the loci (of other types/subtypes) is incomplete due to the fragmented assemblies.

We collected an additional 5812 genes from identified *cas* gene clusters, which do not share similarities with previously identified Cas families. After removing redundant sequences (at 90% a.a. sequence identity) 2836 proteins remain. Among these 2836 proteins, 843 (29.7%) can be assigned to known TIGRFAM or PFAM families, and the remaining (1993; 70.3%) are considered to be putative novel Cas proteins. In the following, we analyzed known TIGRFAM families that have newly discovered associations with the CRISPR–Cas immune system, and novel putative Cas families that we identified by clustering the putative sequences based on their sequence similarities, combined with an examination of their genomic contexts.

### New Cas9 proteins

We focused on the analysis of Cas9 proteins, considering the importance of *cas*9 genes to the recent applications of the CRISPR–Cas systems to genome engineering and gene regulation (22,24,25), even though type II CRISPR–Cas systems are not the prominent form in our collections. From the initial collection of Cas9 proteins, based on similarity searches, we kept only non-redundant sequences (using 90% a.a. sequence identify as the cutoff) that contain the reported catalytic residues: D at position 10 (as in the Cas9 protein from the *S. pyogenes*), and H at position 840. Experiments have shown that when D10 is mutated to A, the target DNA strand non-complementary to the crRNA is not cleaved, and when H840 is mutated to A, the target DNA strand complementary to the crRNA is not cleaved (41). This resulted in 497 Cas9 proteins.

**Figure 1.**
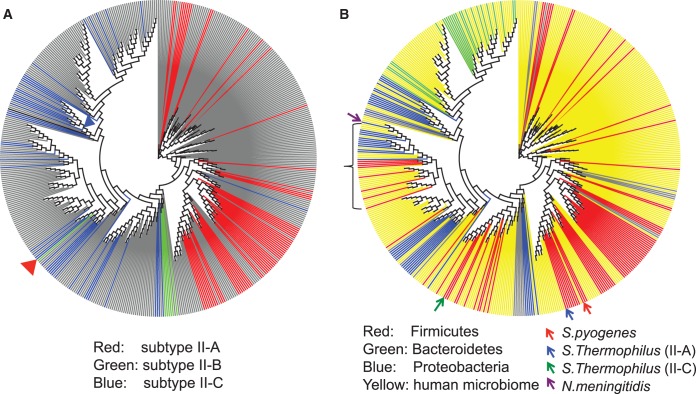
Phylogeny of Cas9 sequences expanded by the HMP datasets, colored by subtype (A) and taxonomy (B). (A) A circular view of the tree with sequences (identified from complete genomes) highlighted based on their subtypes: subtype II-A (red), subtype II-B (green) and subtype II-C (blue); sequences identified from draft genomes or HMP datasets are in gray. This figure shows that Cas9 proteins of different subtypes form separate branches in the phylogenetic tree, with one exception (*Wolinella succinogenes*; NC_005090) highlighted by a red triangle in the graph. (B) A circular view of the Cas9 tree with sequences derived from the HMP datasets highlighted in yellow, and sequences from draft/complete genomes shown in other colors: Firmicutes in red, Proteobacteria in blue, Bacteroidetes in green and others in gray. We highlighted the Cas9 proteins identified from *S. pyogenes*, *S. thermophiles* and *N. meningitides* in the tree with arrows. We note that *S. thermophiles* contains two CRISPR–Cas subtypes: II-A and II-C, and the corresponding Cas9 proteins are grouped with other Cas9 proteins of the same subtype. The bracket highlights a branch including a group of Firmicutes that are more similar to Bacteroidetes than other Firmicutes (see details in Figure 2).

Among the non-redundant set of 497 Cas9 proteins, 176 (35%) were identified from complete or draft bacterial genomes, and many more (321; ∼65%) were identified from the HMP datasets, showing the important contribution of metagenomic sequencing in enriching the sequence diversity of known Cas9 genes, even with thousands of complete bacterial genomes and several thousand draft genomes available. Figure 1A shows that Cas9 proteins belonging to different subtypes of type II CRISPR–Cas systems (subtypes II-A, II-B and II-C) form separate branches in the phylogenetic tree (II-B is the rarest subtype), which is consistent with a previous study (42). One exception is a subtype II-B Cas9 protein (identified from *Wolinella succinogenes*; NC_005090), which is found to be more similar to subtype II-C Cas9 proteins on the tree (see Figure 1A).

**Figure 2.**
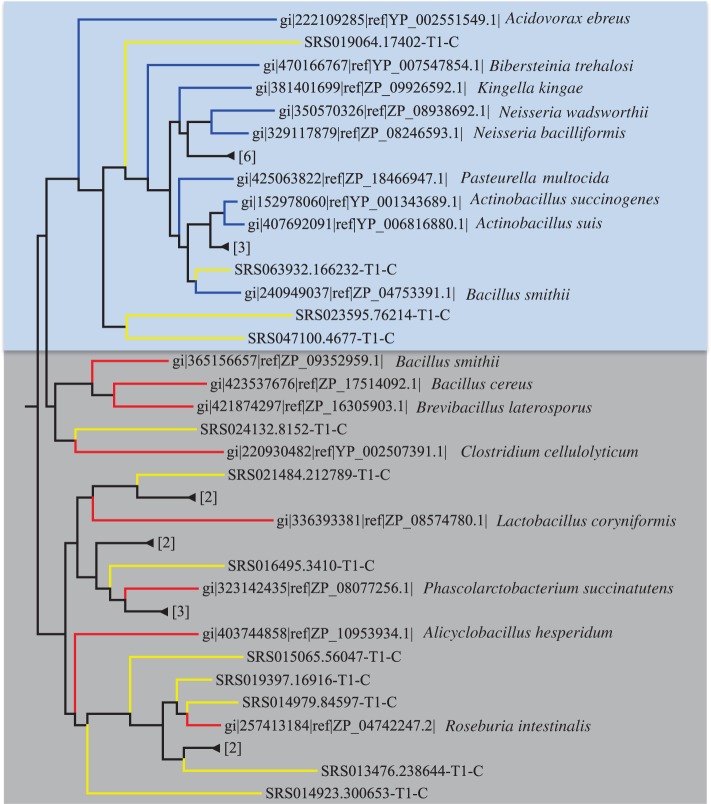
A clade of Cas9 sequences with mixed Firmicutes (highlighted in red) and Proteobacteria (blue) sequences. The species names are shown after the protein IDs. The branches with new Cas9 proteins identified from stool samples are shaded in gray and the branches with new Cas9 proteins identified from oral samples are shaded in blue. Some branches are collapsed for clarity.

Figure 1B shows the phylogenetic tree of Cas9 proteins, colored based on the taxonomic distribution of the sequences and the sources of the sequences: metagenomic Cas9 sequences (in red) are broadly distributed across the tree. The figure shows that sequences from Bacteroidetes form a separate branch, but sequences from Firmicutes and Proteobacteria have various affinities, indicating frequent horizontal transfers of *cas*9 genes among different bacterial species—even among different phyla. In the clade highlighted in Figure 1B, a group of Cas9 proteins identified from Firmicutes species are more similar to those identified from Proteobacteria species than compared to other Firmicutes (see Figure 2 for more details). By contrast, we do not observe sequences segregating by body site in the tree, as shown in Supplementary Figure S1.

The expanded collection of *cas*9 genes has enlarged not only the sequence diversity of Cas9 proteins (see Supplementary Figure S2), but also the diversity of genomic contexts of these genes. We observed co-occurrence of *cas*9 genes (type II signature gene) with types I and III CRISPR–Cas system signature genes. For example, a *cas*9 gene is found in a subtype III-B *cas* loci in *Haliscomenobacter hydrossis* (NC_015510) (Supplementary Figure S3A). In another example, a *cas*9 gene is co-located with subtype I-F signature genes in *Actinobacillus ureae* (NZ_GL831080) (Supplementary Figure S3B).

### Assorted functions associated with the CRISPR–Cas immune system

843 putative Cas proteins can be assigned to known TIGRFAM or PFAM families, but are not currently annotated as CRISPR-associated protein families. There are 21 such families, each containing at least 10 proteins encoded by genes in *cas* loci. Detailed information for these families and their phylogenetic trees are available at our website. Here we focus on a few families.

Among the 21 functions frequently found encoded by genes in *cas* gene clusters, three are related with transposases: 20 proteins are assigned to DDE_Tnp_1 (this family contains transposases for many IS elements, including IS4, IS421, IS5377, IS427, IS402, IS1355 and IS5), 14 proteins are assigned to Transposase_mut (Transposase, Mutator family) and 13 are assigned to Transposase_20 (Transposase IS116/IS110/IS902 family). Other transposase families that are found in our collection of proteins include DDE_Tnp_1_6 (four proteins), DDE_Tnp_ISL3 (two proteins), DDE_Tnp_2 (three proteins), DDE_Tnp_1_3 (two proteins), DDE_Tnp_IS1 (two proteins) and DDE_Tnp_1_4 (one protein). It is likely that most (if not all) of these transposase genes have been randomly inserted into *cas* loci, as about half of the transposase genes are observed on the strand opposite to the *cas* genes. But the actual percent of insertions on the opposite strand differs among different transposase families: 69% (nine out of 13 genes) for the Transposase_20 family (e.g. a transposase_20 gene between 3 043 455 and 3 044 651 of the *Shigella sonnei* Ss046 chromosome, NC_007384, is on the strand opposite to the *cas* genes), 36% for the Transposase_mut family and 43% for the DDE families—perhaps due to some target-site specificity.

19 proteins share significant similarities with the DUF2276 family (PFAM ID: PF10040; an uncharacterized conserved protein family with an average length of 316 a.a.). Out of 19 genes encoding this protein family 17 were collected from bacterial/draft genomes and all these genes themselves had no specific functional annotations. This family shares similarity with Cas6 proteins based on the comparison of their HMMs (http://pfam.sanger.ac.uk/family/PF10040). The proteins we identified share a G-rich motif similar to that of Cas6 proteins: the G-rich motif is on a loop near the C-terminus, with a consensus of GhGxxxxxGhG, where h is hydrophobic and xxxxx should have at least one lysine or arginine (13,43), confirming the similarity between DUF2276 and the Cas6 family. Genomic contexts of the genes encoding for DUF2276 proteins, however, are different from Cas6: eight genes (out of 19) encoding DUF2276 proteins were found next to the *cas* gene encoding protein VVA1548 (TIGR02620; the gene symbol is csx16 provided by TIGRFAM database) (but the other gene neighbor varies); however, Cas6 was found to be next to Cas VVA1548 only in 34 out of 3958 (∼0.86%) genomes (and if a DUF2276 gene is next to Cas VVA1548, its other neighbor is likely to be Cas RAMPs).

**Figure 3.**
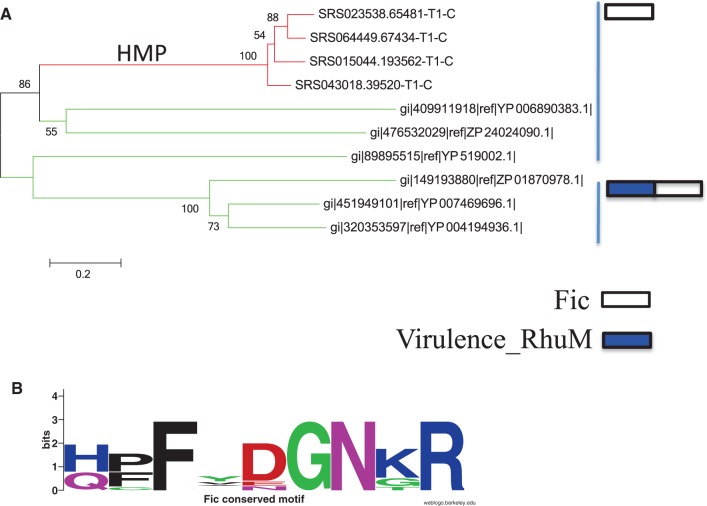
A phylogenetic tree of the identified Fic toxin proteins (sequences identified from the HMP datasets are highlighted) (A). Some of these proteins contain only a Fic domain, whereas others include a Virulence_RhuM domain, as shown on the right. (B) The sequence logo of the region corresponding to the known Fic motif. Only two genes gi|476532029|ref|ZP_24024090.1 and gi|409911918|ref|YP_006890383.1 from bacterial genomes share the exact motif of the Fic/DOC family: HPFXXGNG.

It has been reported that *cas* loci often include genes that encode toxins (44). We found 10 genes that encode toxins belonging to the Fic/DOC family (PF02661 in PFAM); the average length of this family is 256 a.a.. Among these 10, four sequences were from the HMP datasets (Figure 3). All the *cas* loci containing this gene from complete or draft genomes have *cas*3 gene, a feature of type I CRISPR–Cas systems (12), suggesting that the CRISPR–Cas systems containing this toxin-encoding gene belong to type I. Two (ZP_24024090.1 and YP_006890383.1 from bacterial genomes) of the 10 sequences contain the Fic/DOC motif HPFxxGNG, whereas the other eight sequences contain a slightly different motif (see Figure 3B for a sequence logo). We also checked the genomic contexts and found that, in *Geobacter sulfurreducens* (NC_017454), the Fic-encoding gene is located next to a gene that encodes an antitoxin (YP_006890382.1; the hmmsearch shows that it is similar to TIGRFAM family TIGR02612). This is an example of *cas* loci with both toxin and antitoxin, indicating potential coupling between immunity and dormancy/suicide defense systems in prokaryotes, and is the first-reported antitoxin-encoding gene reported in *cas* loci (44).

**Figure 4.**
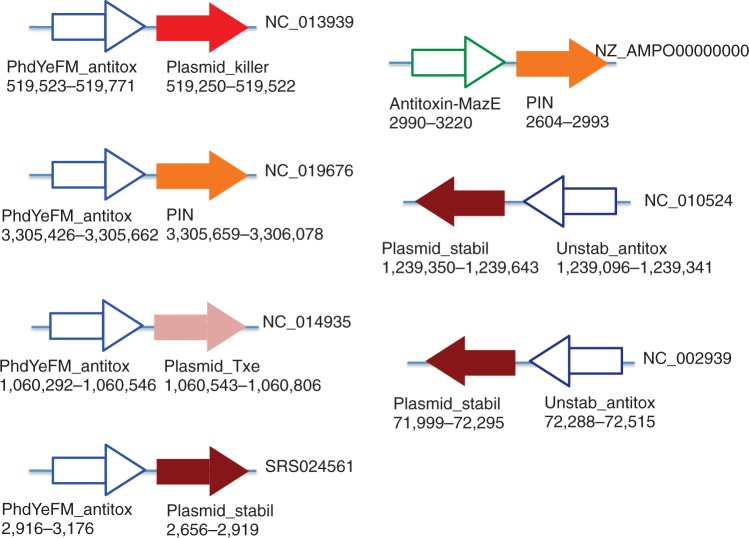
Examples of the toxin–antitoxin systems found in *cas* loci. Antitoxin genes are shown as open arrows, and toxin genes as filled arrows. Genes are orientated such that known *cas* genes in the same locus are transcribed from left to right (thus, a toxin/antitoxin gene oriented from right to left is on the opposite strand of the *cas* genes).

Additionally, we found three other antitoxin families in CRISPR–Cas loci: PhdYeFM_antitox (eight sequences), Antitoxin-MazE (eight sequences) and Unstab_antitox (three sequences). For all three antitoxin families, we found *cas* loci containing both antitoxin and toxin genes, providing further evidence that immunity and dormancy/suicide defense systems are coupled in bacteria. For example, the *cas* locus in the genome of *Nostoc* sp. PCC 7107 contains an antitoxin gene belonging to the PhdYeFM_antitox family (YP_007050591.1 between 3 305 426 and 3 305 662 bp), and a toxin gene (between 3 305 659 and 3 306 078) encoding a protein (YP_007050592.1) similar to the PIN family: the majority of PIN-domain proteins found in prokaryotes are the toxic components of toxin–antitoxin operons, providing a control mechanism that helps free-living prokaryotes cope with nutritional stress (45). See Figure 4 for more examples of toxin–antitoxin gene pairs in *cas* loci. In most of the cases we found, the antitoxin genes are located upstream of their cognate toxin genes—such an organization appears to promote production of the antitoxins at higher levels than that of their cognate toxins (46)—and most of them are located on the same strand as the *cas* genes, with exceptions (as shown in Figure 4). We note that toxin–antitoxin systems were only found in type I CRISPR–Cas systems in our collection (see Supplementary Figure S4).

Another interesting function we observed is 23S_rRNA_IVP, a family consisting of bacterial proteins encoded within an intervening sequence present within some 23S rRNA genes (47). We found 18 genes in *cas* loci that encode proteins belonging to this family. We observed a conserved motif with a consensus of GxxRxxxSxxxNxxE, which was also observed in the alignment of Xcc0516 homologs built by Lin *et al.* (48). By analyzing the genomic contexts of this gene, we found that 23S_rRNA_IVP genes often co-occur with genes encoding nucleases such as Cas4 (exonuclease) or/and Cas6 (endoribonucleases). 23S_rRNA_IVP genes are found in all three types of CRISPR–Cas systems (including I-B, I-E, II-A and III-B). For example, one gene-encoding 23S_rRNA_IVP (YP_007081119.1 between 2 412 782 and 2 413 162 bp) is found between a gene-encoding Cmr3 (YP_007081118.1 between 2 411 515 and 2 412 696 bp; *cmr*3 genes are unique to subtype III-B CRISPR–Cas systems) and a gene-encoding Cmr2 (YP_007081120.1 between 2 413 168 and 2 416 287) in *Pleurocapsa sp. PCC 7327* chromosome (NC_019689).

### Novel Cas families

**Figure 5.**
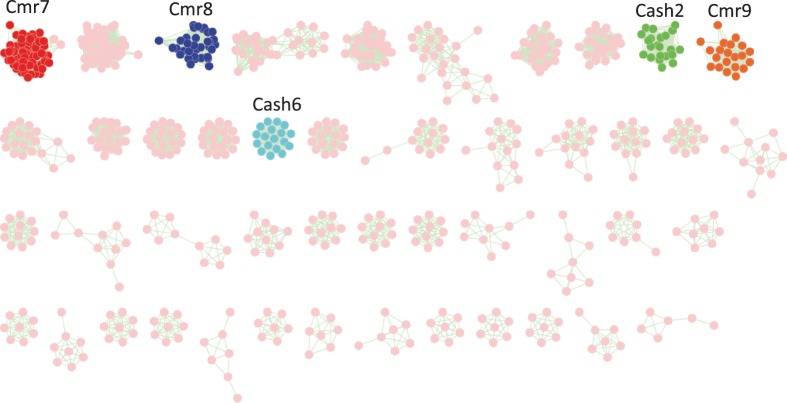
MCL clustering of 1993 putative Cas proteins. Several families are highlighted in the figure, including Cmr7 (the largest family containing 69 proteins), Cmr8 containing 34 and Cmr9 containing 21; Cash2 containing 22 proteins. We only show a few large families in this figure—detailed families are shown in the Supplementary Figure S5.

We clustered the remaining 1993 putative Cas proteins (1266 proteins were predicted from bacterial/draft genomes and 727 from the HMP datasets) that were not assigned to known Cas proteins or other TIGRFAM/PFAM families, using the MCL approach, hoping to infer putative novel Cas protein families. Overall, we obtained 31 clusters, each containing at least five proteins with at most 50% identity (see Figure 5 and Supplementary Figure S5); smaller clusters (including 844 singletons) were not considered in further analyses. We further confirmed the associations of the putative Cas proteins by checking the genomic contexts of their genes: we found that genes in the same cluster of putative Cas proteins tend to share similar neighboring *cas* genes. We note that weak similarities were observed between seven putative novel families and known Cas proteins based on BLASTP searches, using loose criteria (*E*-value ≤ 1*e* – 5 and sequence identity 30%), and by comparisons of their profile HMMs; these seven clusters were excluded from our collection of novel Cas families. As a further quality control, we searched the remaining 24 clusters against AntiFam (which collects suspicious ORFs) and CRISPR array repeats, and no matches were found; we also checked reads coverage for the *cas* genes identified from HMP datasets, all indicating a low chance of misassemblies (see Supplementary Figure S6). We therefore only consider the remaining 24 clusters as putative novel Cas protein families.

We assigned the types (and subtypes) to the Cas families based on genomic context analyses (see Supplementary Table S3 for details). We named three families Cmr7, Cmr8 and Cmr9 (as they are found to be in type III-B CRISPR–Cas systems), and the remaining as cash1–cash21 (in which ‘h’ is for ‘hmp’). Taxonomic assignments of our novel Cas proteins by MEGAN4 (49) show a taxonomic distribution that is typical to human microbiomes (see Supplementary Figure S7), with more genes assigned to Actinomycetaceae, Coriobacteriaceae, Prevotella [found in all nine oral subsites including soft palate and tonsils (50)] at family level, and *Leptotrichia buccalis* [which is commonly found in subgingival plaque microbiome (51)] at species level. Below we present detailed analyses of several of these novel families.

**Figure 6.**
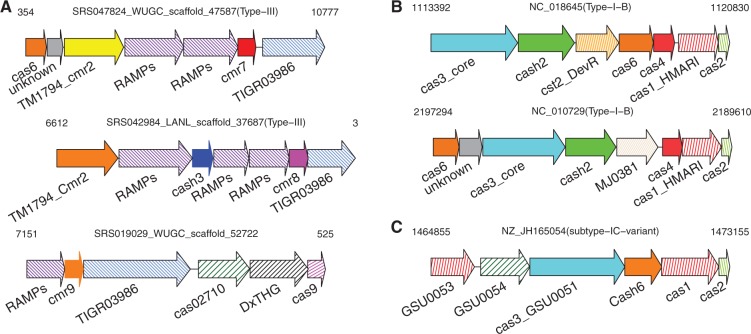
Representative genomic contexts for selected putative novel *cas* genes. (A) *cmr*7 genes, *cmr*8 genes and *cmr*9 do not share sequence similarity, but have similar genomic contexts: 96% of the *cmr*7 genes are next to a RAMP gene (and 40 also have a flanking TIGR03986); 82% of the *cmr*8 genes and 95% of the *cmr*9 genes are found between RAMP and TIGR03986 genes. (B) About half of the *cash*2 genes are located between Cas3_core and Cst2_DevRas, while another half of them have Cas3_core and MJ0381 proteins (non-Cas protein) as neighbors. (C) A *cash*6 gene that shares similar genomic contexts as *cas*4 genes.

Three families of novel *cas* genes share similar genomic contexts, and are found to co-occur with type III-B specific genes (and therefore are likely to be involved in bacterial defense against RNA molecules), so we named them Cmr7, Cmr8 and Cmr9 (Cmr families are unique to type III-B CRISPR–Cas systems). Genes of *cmr*7 *and cmr*8 are only found in oral microbiomes (not in stool samples) and only one gene of *cmr*9 family is identified from a stool sample. The largest novel family, Cmr7, has 69 non-redundant putative Cas proteins of average length 194 a.a.; Cmr8 contains 34 proteins of average length 142 a.a. and Cmr9 contains with 21 proteins of average length 138 a.a. 61 (88%) of the *cmr*7 genes were collected from HMP data (mostly the tongue dorsum samples) and the remaining eight sequences were identified from either complete or draft genomes, all belonging to the Actinobacteria phylum. Genes of this family are found between RAMP (66 cases, among which 12 are *cas_cyan_RAMP* genes) and TIGR03986 genes (40 cases; TIGR03986 is described as a ‘CRISPR-associated protein’ but without further details); a representative genomic context, SRS047824_WUGC_scaffold_47587, is shown in Figure 6A. Similarly, most of the genes in Cmr8 (30, 88%) and Cmr9 (20, 95%) are located between RAMP and TIGR03986 genes, or two RAMP genes (see an example in Figure 6A). These three families of *cas* genes are exclusively found in *cas* loci (no additional proteins outside of our collection were identified by searching their HMMs against bacterial genomes for Cmr7 and Cmr8; and only two additional proteins were found for Cmr9). The lack of sequence similarity among these three families was further confirmed by a comparison of their HMMs by HHalign (52). All suggests that while these three novel families share a similar CRISPR-associated function, they do not share sequence similarity.

Family Cash2 contains 22 proteins of average length 404 a.a.. In this family, 13 (∼59%) of the genes were collected from bacterial/draft genomes. From this family, 21 (95%) of the genes are located next to *cas*3*_core* genes: *cas*3*_core* genes encode nucleases that degrade invader DNA in type I CRISPR–Cas systems. The genes downstream of *cash*2 genes are Cst2_DevRas members, as in *Desulfobacula toluolica* (NC_018645), or MJ0381 (TIGR01875; CRISPR-associated auto-regulator DevR family) as in *Porphyromonas gingivalis* (NC_010729) (see Figure 6B). We note that Cst2 family proteins [also annotated as Cas7 (12)] are found in type I CRISPR–Cas systems, and their function was originally described as COG1857 and COG3649 (both are uncharacterized proteins predicted to be involved in DNA repair) (http://www.ncbi.nlm.nih.gov/COG) (53). Similarity searches using the HMM of Cash2 proteins against bacterial proteins did not recruit any additional proteins, nor any encoded by genes located outside of *cas* loci, indicating that *cash*2 genes are exclusively found in *cas* loci. As *cash*2 genes tend to be found together with *cas* genes from the type I CRISPR–Cas system, *cash*2 genes are likely to be involved in the type I CRISPR–Cas systems, which provide defense against DNA molecules.

Family Cash3 contains 20 proteins of average length 158 a.a., all identified from the HMP datasets: nine proteins from supragingival plaque microbiomes and nine proteins from tongue dorsum microbiomes. This family is likely to be involved in type III CRISPR–Cas systems, since all *cash*3 genes are located between RAMPs genes, and type III signature genes are found in the neighborhood. Proteins of this family share a conserved motif that is phenylalanine and tyrosine rich (Supplementary Figure S8A), and are likely to form helical structures [as predicted by I-TASSER (54)] and bind DNA molecules [as predicted by iDBPs server (55)] (see Supplementary Figure S8B for a model of the complex structure).

The last example Cash6, is found associated with the CRISPR–Cas subtype IC-variant, and shares similar genomic contexts with *cas*4 genes; for example, Figure 6C shows the *cash*6 gene in *Propionibacterium avidum* (NZ_JH165054.1), which has a subtype IC-variant signature gene (GSU0054 or GSU0053) (33) in the neighborhood (see Figure 6C). We hypothesize that Cash6 is likely to be a non-homologous replacement of the known Cas4 family: these families share similar genomic contexts but not detectable sequence similarity.

### Oral microbiomes carry more *cas* genes than stool samples

The microbial communities collected from healthy volunteers are particularly diverse in oral and stool habitats (28). Previous studies, including one of our own (27), investigated the CRISPR composition of human microbial communities, especially in the oral habitat, focusing on CRISPR arrays (56,57). In this study, we examined the frequency of *cas* genes in the microbial communities of each body habitat: not surprisingly, most *cas* genes were collected from the two most complex body sites: 27 532 (20.5%) *cas* genes from stool samples and 100 721 (75%) from the three sub-oral sites (tongue dorsum, buccal mucosa, supragingival plaque). Most Cas families are found in both stool and oral microbiomes, whereas a few are found exclusively (or dominantly) in a specific body site (for example, we only identified Cmr7 and Cmr8 in oral microbiomes, as mentioned above; and 88% of the CasA_cse1 proteins are found in stool datasets).

**Figure 7.**
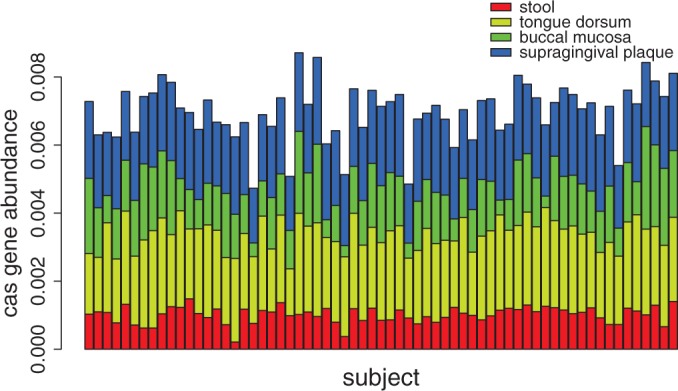
Relative abundances of *cas* genes in the gut and oral microbial communities across 65 individuals.

We further selected samples from 65 subjects, in all of whom *cas* genes were found in all stool and sub-oral habitats. As shown in Figure 7, the percentage of *cas* genes (see Methods section) in stool samples was remarkably lower than the percentage in tongue dorsum and supragingival plaque (*P*-value < 2.2*e* – 16, paired *t*-test). Additionally, within the oral habitat, the *cas* gene percentage in tongue dorsum samples was significantly greater than the percentage in supragingival plaque samples (*P*-value = 8.939*e* – 6, paired *t*-test), while the percentage in buccal mucosa samples was significantly lower than in supragingival plaque (*P*-value = 1.158*e* – 15, paired *t*-test). This trend of *cas* gene distributions is consistent with our analysis of invasive mobile genetic elements: we found that oral sites carry more invasive MGEs as compared to stool samples (58). However, there is not a correlation (as measured by Pearson correlation coefficient) between the abundances of *cas* genes in stool samples and in oral sites within individuals, indicating that the abundance of *cas* genes in the oral habitat does not mean a higher abundance of the *cas* genes in the stool habitat of the same subject.

## DISCUSSION

Using an approach that combines similarity searches and genomic context analysis, we were able to significantly expand the collection of known *cas* genes, and also to identify putative novel Cas protein families. Metagenomics projects have resulted in a daunting number of hypothetical proteins, and one of the computational challenges raised by metagenomics is to annotate these hypothetical proteins. Here we show that targeted analyses of general metagenomic datasets can be rewarding, resulting in the identification of new instances of known Cas families, and novel Cas proteins that may play various functions in the bacterial immune systems.

Our analysis of *cas*9 genes suggests that there are horizontal transfers of *cas*9 genes between different species, even across different phyla. It will be interesting to test if the transfers involve whole *cas* loci or individual *cas* genes, by comparing the evolutional history of the different *cas* genes. Another direction we will take is to study the diversity of *cas*9 genes and correlate this diversity with the invasive DNA elements that they attack, hoping to identify *cas*9 genes that have different preferences for PAMs (Protospacer Adjacent Motifs), which will enhance cas9’s applications in genome engineering.

Bacteria have developed various types of defense mechanisms to protect against invaders. Emerging evidence suggests these different mechanisms can be coupled, including the coupling of immunity and dormancy/suicide defense systems (44,59), to orchestrate a cell’s response to attack. Better defining functions associated with each of these defense units, such as our finding of numerous toxin and antitoxin genes within CRISPR–Cas loci, will continue to improve our understanding of bacterial immunity.

We have used different approaches to confirm that the novel Cas families we identified are not similar to known families. It is still possible that some of our novel Cas families (especially small families with relatively few sequences) will turn out to be remote homologs of known Cas families as more sequences become available, bridging the sequence-similarity gap between our families and known ones, or when new computational tools for more sensitive similarity detection are available. However, we think our collection of novel families (even if some of them are actually sub-families to known ones) is still a valuable addition to the current collection of Cas families.

## SUPPLEMENTARY DATA

Supplementary Data are available at NAR Online.

Supplementary Data

## References

[1] Barrangou R, Fremaux C, Deveau H, Richards M, Boyaval P, Moineau S, Romero DA, Horvath P (2007). CRISPR provides acquired resistance against viruses in prokaryotes. Science.

[2] Garneau JE, Dupuis ME, Villion M, Romero DA, Barrangou R, Boyaval P, Fremaux C, Horvath P, Magadan AH, Moineau S (2010). The CRISPR/Cas bacterial immune system cleaves bacteriophage and plasmid DNA. Nature.

[3] Horvath P, Barrangou R (2010). CRISPR/Cas, the immune system of bacteria and archaea. Science.

[4] Marraffini LA, Sontheimer EJ (2010). CRISPR interference: RNA-directed adaptive immunity in bacteria and archaea. Nat. Rev. Genet..

[5] Semenova E, Jore MM, Datsenko KA, Semenova A, Westra ER, Wanner B, van der Oost J, Brouns SJ, Severinov K (2011). Interference by clustered regularly interspaced short palindromic repeat (CRISPR) RNA is governed by a seed sequence. Proc. Natl Acad. Sci. USA.

[6] Sorek R, Kunin V, Hugenholtz P (2008). CRISPR–a widespread system that provides acquired resistance against phages in bacteria and archaea. Nat. Rev. Microbiol..

[7] van der Oost J, Jore MM, Westra ER, Lundgren M, Brouns SJ (2009). CRISPR-based adaptive and heritable immunity in prokaryotes. Trends. Biochem. Sci..

[8] Deltcheva E, Chylinski K, Sharma CM, Gonzales K, Chao Y, Pirzada ZA, Eckert MR, Vogel J, Charpentier E (2011). CRISPR RNA maturation by trans-encoded small RNA and host factor RNase III. Nature.

[9] Deveau H, Barrangou R, Garneau JE, Labonte J, Fremaux C, Boyaval P, Romero DA, Horvath P, Moineau S (2008). Phage response to CRISPR-encoded resistance in *Streptococcus thermophilus*. J. Bacteriol..

[10] Jansen R, Embden JD, Gaastra W, Schouls LM (2002). Identification of genes that are associated with DNA repeats in prokaryotes. Mol. Microbiol..

[11] Grissa I, Vergnaud G, Pourcel C (2007). The CRISPRdb database and tools to display CRISPRs and to generate dictionaries of spacers and repeats. BMC Bioinformatics.

[12] Makarova KS, Haft DH, Barrangou R, Brouns SJ, Charpentier E, Horvath P, Moineau S, Mojica FJ, Wolf YI, Yakunin AF (2011). Evolution and classification of the CRISPR-Cas systems. Nat. Rev. Microbiol..

[13] Haft DH, Selengut J, Mongodin EF, Nelson KE (2005). A guild of 45 CRISPR-associated (Cas) protein families and multiple CRISPR/Cas subtypes exist in prokaryotic genomes. PLoS Comput. Biol..

[14] Brouns SJ, Jore MM, Lundgren M, Westra ER, Slijkhuis RJ, Snijders AP, Dickman MJ, Makarova KS, Koonisn EV, van der Oost J (2008). Small CRISPR RNAs guide antiviral defense in prokaryotes. Science.

[15] Marraffini LA, Sontheimer EJ (2009). Invasive DNA, chopped and in the CRISPR. Structure.

[16] Beloglazova N, Brown G, Zimmerman MD, Proudfoot M, Makarova KS, Kudritska M, Kochinyan S, Wang S, Chruszcz M, Minor W (2008). A novel family of sequence-specific endoribonucleases associated with the clustered regularly interspaced short palindromic repeats. J. Biol. Chem..

[17] Hale CR, Majumdar S, Elmore J, Pfister N, Compton M, Olson S, Resch AM, Glover CV, Graveley BR, Terns RM (2012). Essential features and rational design of CRISPR RNAs that function with the Cas RAMP module complex to cleave RNAs. Mol. Cell.

[18] Marraffini LA, Sontheimer EJ (2008). CRISPR interference limits horizontal gene transfer in staphylococci by targeting DNA. Science.

[19] Chylinski K, Le Rhun A, Charpentier E (2013). The tracrRNA and Cas9 families of type II CRISPR-Cas immunity systems. RNA Biol..

[20] Gasiunas G, Barrangou R, Horvath P, Siksnys V (2012). Cas9-crRNA ribonucleoprotein complex mediates specific DNA cleavage for adaptive immunity in bacteria. Proc. Natl Acad. Sci. USA.

[21] Mali P, Yang L, Esvelt KM, Aach J, Guell M, Dicarlo JE, Norville JE, Church GM (2013). RNA-Guided human genome engineering via Cas9. Science.

[22] Cong L, Ran FA, Cox D, Lin S, Barretto R, Habib N, Hsu PD, Wu X, Jiang W, Marraffini LA (2013). Multiplex genome engineering using CRISPR/Cas systems. Science.

[23] Dicarlo JE, Norville JE, Mali P, Rios X, Aach J, Church GM (2013). Genome engineering in *Saccharomyces cerevisiae* using CRISPR-Cas systems. Nucleic Acids Res..

[24] Chang N, Sun C, Gao L, Zhu D, Xu X, Zhu X, Xiong JW, Xi JJ (2013). Genome editing with RNA-guided Cas9 nuclease in Zebrafish embryos. Cell Res..

[25] Qi LS, Larson MH, Gilbert LA, Doudna JA, Weissman JS, Arkin AP, Lim WA (2013). Repurposing CRISPR as an RNA-guided platform for sequence-specific control of gene expression. Cell.

[26] Sorokin VA, Gelfand MS, Artamonova II (2010). Evolutionary dynamics of clustered irregularly interspaced short palindromic repeat systems in the ocean metagenome. Appl. Environ. Microbiol..

[27] Rho M, Wu YW, Tang H, Doak TG, Ye Y (2012). Diverse CRISPRs evolving in human microbiomes. PLoS Genet..

[28] The HMP Consortium (2012). Structure, function and diversity of the healthy human microbiome. Nature.

[29] The HMP Consortium (2012). A framework for human microbiome research. Nature.

[30] Haft DH, Selengut JD, White O (2003). The TIGRFAMs database of protein families. Nucleic Acids Res..

[31] Punta M, Coggill PC, Eberhardt RY, Mistry J, Tate J, Boursnell C, Pang N, Forslund K, Ceric G, Clements J (2012). The Pfam protein families database. Nucleic Acids Res..

[32] Eddy SR (2011). Accelerated Profile HMM Searches. PLoS Comput. Biol..

[33] Makarova KS, Aravind L, Wolf YI, Koonin EV (2011). Unification of Cas protein families and a simple scenario for the origin and evolution of CRISPR-Cas systems. Biol. Direct..

[34] Li W, Godzik A (2006). Cd-hit: a fast program for clustering and comparing large sets of protein or nucleotide sequences. Bioinformatics.

[35] Kurokawa K, Itoh T, Kuwahara T, Oshima K, Toh H, Toyoda A, Takami H, Morita H, Sharma VK, Srivastava TP (2007). Comparative metagenomics revealed commonly enriched gene sets in human gut microbiomes. DNA Res..

[36] Enright AJ, Van Dongen S, Ouzounis CA (2002). An efficient algorithm for large-scale detection of protein families. Nucleic Acids Res..

[37] Shannon P, Markiel A, Ozier O, Baliga NS, Wang JT, Ramage D, Amin N, Schwikowski B, Ideker T (2003). Cytoscape: a software environment for integrated models of biomolecular interaction networks. Genome Res..

[38] Edgar RC (2004). MUSCLE: a multiple sequence alignment method with reduced time and space complexity. BMC Bioinformatics.

[39] Price MN, Dehal PS, Arkin AP (2009). FastTree: computing large minimum evolution trees with profiles instead of a distance matrix. Mol. Biol. Evol..

[40] Hedlund J, Jornvall H, Persson B (2010). Subdivision of the MDR superfamily of medium-chain dehydrogenases/reductases through iterative hidden Markov model refinement. BMC Bioinformatics.

[41] Jinek M, Chylinski K, Fonfara I, Hauer M, Doudna JA, Charpentier E (2012). A programmable dual-RNA-guided DNA endonuclease in adaptive bacterial immunity. Science.

[42] Chylinski K, Le Rhun A, Charpentier E (2013). The tracrRNA and Cas9 families of type II CRISPR-Cas immunity systems. RNA Biol..

[43] Carte J, Wang R, Li H, Terns RM, Terns MP (2008). Cas6 is an endoribonuclease that generates guide RNAs for invader defense in prokaryotes. Genes Dev..

[44] Koonin EV, Makarova KS (2013). CRISPR-Cas: Evolution of an RNA-based adaptive immunity system in prokaryotes. RNA Biol..

[45] Arcus VL, McKenzie JL, Robson J, Cook GM (2011). The PIN-domain ribonucleases and the prokaryotic VapBC toxin-antitoxin array. Protein Eng. Des. Sel..

[46] Yamaguchi Y, Park JH, Inouye M (2011). Toxin-antitoxin systems in bacteria and archaea. Annu. Rev. Genet..

[47] Ralph D, McClelland M (1993). Intervening sequence with conserved open reading frame in eubacterial 23S rRNA genes. Proc. Natl Acad. Sci. USA.

[48] Lin LY, Ching CL, Chin KH, Chou SH, Chan NL (2006). Crystal structure of the conserved hypothetical cytosolic protein Xcc0516 from *Xanthomonas campestris* reveals a novel quaternary structure assembled by five four-helix bundles. Proteins.

[49] Huson DH, Mitra S, Ruscheweyh HJ, Weber N, Schuster SC (2011). Integrative analysis of environmental sequences using MEGAN4. Genome Res..

[50] Aas JA, Paster BJ, Stokes LN, Olsen I, Dewhirst FE (2005). Defining the normal bacterial flora of the oral cavity. J. Clin. Microbiol..

[51] Paster BJ, Boches SK, Galvin JL, Ericson RE, Lau CN, Levanos VA, Sahasrabudhe A, Dewhirst FE (2001). Bacterial diversity in human subgingival plaque. J. Bacteriol..

[52] Remmert M, Biegert A, Hauser A, Soding J (2012). HHblits: lightning-fast iterative protein sequence searching by HMM-HMM alignment. Nat. Methods.

[53] Tatusov RL, Fedorova ND, Jackson JD, Jacobs AR, Kiryutin B, Koonin EV, Krylov DM, Mazumder R, Mekhedov SL, Nikolskaya AN (2003). The COG database: an updated version includes eukaryotes. BMC Bioinform..

[54] Roy A, Kucukural A, Zhang Y (2010). I-TASSER: a unified platform for automated protein structure and function prediction. Nat. Protoc..

[55] Nimrod G, Schushan M, Szilagyi A, Leslie C, Ben-Tal N (2010). iDBPs: a web server for the identification of DNA binding proteins. Bioinformatics.

[56] Pride DT, Salzman J, Relman DA (2012). Comparisons of clustered regularly interspaced short palindromic repeats and viromes in human saliva reveal bacterial adaptations to salivary viruses. Environ. Microbiol..

[57] Pride DT, Sun CL, Salzman J, Rao N, Loomer P, Armitage GC, Banfield JF, Relman DA (2011). Analysis of streptococcal CRISPRs from human saliva reveals substantial sequence diversity within and between subjects over time. Genome Res..

[58] Zhang Q, Rho M, Tang H, Doak TG, Ye Y (2013). CRISPR-Cas systems target a diverse collection of invasive mobile genetic elements in human microbiomes. Genome Biol..

[59] Anantharaman V, Makarova KS, Burroughs AM, Koonin EV, Aravind L (2013). Comprehensive analysis of the HEPN superfamily: identification of novel roles in intra-genomic conflicts, defense, pathogenesis and RNA processing. Biol. Direct..

