# Supplementary material for: Expanding the catalog of cas genes with metagenomes
Source: Nucleic Acids Res. 2013 Dec 5;42(4):2448–59. doi: 10.1093/nar/gkt1262 (PMC3936711; doi:10.1093/nar/gkt1262)
Supplement: Supplementary Data [file supp_42_4_2448__index.html]

Expanding the catalog of cas genes with metagenomes — Expanding the catalog of cas genes with metagenomes — Supplementary Data 

# Expanding the catalog of *cas* genes with metagenomes

## Supplementary Data

files

**Files in this Data Supplement:**

- Supplementary Data - docx file
