# Supplementary material for: Expanding the catalog of cas genes with metagenomes
Source: Nucleic Acids Res. 2013 Dec 5;42(4):2448–59. doi: 10.1093/nar/gkt1262 (PMC3936711; doi:10.1093/nar/gkt1262)
Supplement: Supplementary Data [file supp_gkt1262_nar-02040-z-2013-File009.docx]

**This document contains Supplementary Tables S1-S3 and Figures S1-S8**

Expanding the Catalog of *cas* Genes by Metagenomes

Quan Zhang, Thomas G. Doak and Yuzhen Ye

**Table S1.** The quantification of four distinct sets of genes collected from bacterial genomes/HMP contigs.

| Gene name | Bacterial genomes | Draft genomes | HMP contigs |
| --- | --- | --- | --- |
| Cas1 | 1,444 | 2,515 | 25,837 |
| Cas2 | 1,253 | 2,153 | 15,224 |
| Cas3 | 1,197 | 2,054 | 8,363 |
| Cas6 | 656 | 593 | 2,709 |
| Cas9/csn1 | 199 | 449 | 6,382 |
| Cas9/csx12 | 13 | 23 | 46 |
| Cas10/cmr2 | 245 | 143 | 606 |
| Cas10/ csm1 | 181 | 130 | 1,972 |
| Cas10/ csx11 | 37 | 3 | 0 |
| Novel genes | 785 | 481 | 727 |

**Table S2.** Relative abundance of core *cas* genes in human microbiomes.

| Cas | | Number of genes (% among 131,117) |
| --- | --- | --- |
| Cas1 | Core Cas1 | 14,710 (11.22%) |
|  | Cas1 in specific subtype | 111,27 (8.49%) |
| Cas2 | Cas2  Cas2 CT1978 | 13,714 (10.46%)  1,510 (1.15%) |
| Cas3 | Core Cas3 | 4,116 (3.14%) |
|  | cas3-type HD domain | 3,660 (2.79%) |
|  | Cas3 in specific | 587 (0.45%) |
| Cas9 | Csn1 | 6,382 (4.87%) |
|  | Csx12 | 46 (0.04%) |
| Cas10 | TM1794_Cmr2 | 606 (0.46%) |
|  | TM1811_Csm1 | 1,972 (1.50%) |

**Table S3.** Details for 24 novel Cas families.

| Cluster ID | *cas* loci only? | context | #(sequences) | | Len (aa) | Subtype | #seq assigned to types | #seq with remote homolog |
| --- | --- | --- | --- | --- | --- | --- | --- | --- |
|  |  |  | 90%-nr | 50%-nr |  |  |  |  |
| cmr7 | Yes | RAMPs--TIGR03986/RAMP | 69 | 15 | 194 | III-B; III | 20  (29%) | RAMPs: 3 |
| cmr8 | Yes | RAMPs--TIGR03986/RAMP | 34 | 7 | 142 | III; III-B | 12  (35%) | Cas1:1 |
| cmr9 | No (2)^a^ | RAMPs--TIGR03986/RAMP | 21 | 9 | 138 | III-U; III-B | 17  (81%) | - |
| cash1 | No (18) |  | 23 | 14 | 216 | I; I-A; I-B;  III; III-A; III-B | 23  (100%) | Cas6: 1  DxTHG: 3 |
| cash2 | Yes | cas3-- MJ0381/ Cst2_DevR | 22 | 12 | 404 | I; I-B | 22  (100%) | - |
| cash3 | Yes | RAMP--RAMPs | 20 | 5 | 158 | III | 5  (25%) | - |
| cash4 | No (9) | RAMPs--RAMPs/ Cmr4 | 18 | 10 | 134 | III-B  I-A; I-B; I-C-variant | 18  (100%) | Cmr5:4 |
| cash5 | No (1) | RAMP—RAMPs | 18 | 13 | 154 | III; III-A; III-B; III-U | 17  (94%) | TIGR03984:2 |
| cash6 | No (3) | cas1-- cas3_GSU0051 | 18 | 10 | 348 | I-C-variant | 18  (100%) | - |
| cash7 | No (5) |  | 13 | 10 | 261 | III; III-A; III-B  I-A; I-B | 13  (100%) | - |
| cash8 | No (1) | MJ0381--cas3 | 12 | 10 | 549 | I; I-B | 11  (92%) | DxTHG:1 |
| csh9 | No (2) | cas6--TM1794_Cmr2 | 11 | 6 | 411 | III-B; III | 10  (91%) | - |
| cash10 | No (34) | cas3--Cse1 | 11 | 8 | 181 | I-E | 11  (100%) | - |
| cash11 | Yes | Cas1--Csn1 | 10 | 8 | 304 | II; II-A | 10  (100%) | Cas9/Csn1:1 |
| cash12 | No (2) | cas6/ TIGR02619 --RAMPs | 10 | 7 | 218 | III; III-A;III-B; III-U | 10  (100%) | - |
| cash13 | No (5) |  | 9 | 8 | 99 | III-B; III-U; III-A | 8  (89%) | - |
| cash14 | No (4) |  | 8 | 5 | 565 | I-U; I-B  III-U | 3  (38%) | - |
| cash15 | Yes | Cst2_DevR--cas3 | 7 | 5 | 585 | I; III-B | 7  (100%) | - |
| cash16 | No (5) | RAMP-- RAMP | 7 | 6 | 144 | III; III-A; III-U | 6  (86%) | - |
| cash17 | No (2) |  | 7 | 7 | 663 | III-B | 4  (57%) | - |
| cash18 | Yes | cas6--MJ0381 | 7 | 6 | 245 | I; I-B | 5  (71%) | - |
| cash19 | Yes | TM1794_Cmr2 --cas6 | 7 | 5 | 226 | III; III-B | 6  (86%) | - |
| cash20 | Yes | RAMP—RAMP | 6 | 5 | 148 | III; III-B | 6  (100%) | Cmr5:1 |
| cash21 | No (1) |  | 5 | 5 | 545 | III-B | 1  (20%) | - |

^a^: number of sequences found outside of *cas* loci that are similar to the cas family sequences


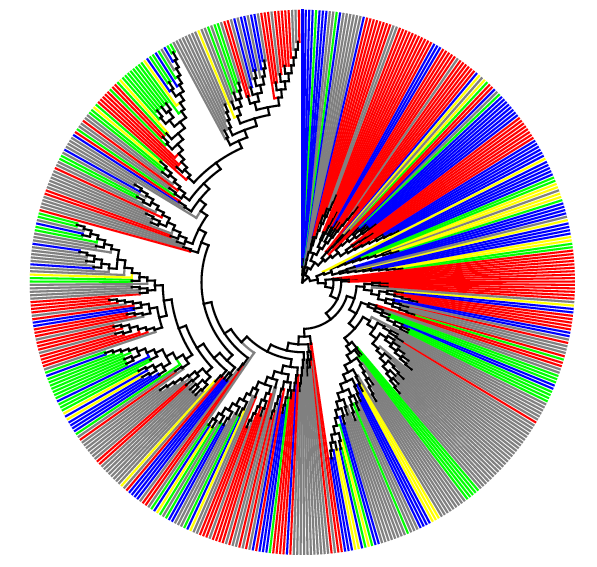


**Figure S1**. A circular view of the tree with metagenomic sequences highlighted based on their body site sources: stool (red), tongue dorsum (blue), and supragingival plaque (green); sequences from complete or draft genomes are in gray.

**Figure S2**. Sequence diversity of Cas9 proteins, as shown in several selected branches. Only the regions around the two active sites (D10 and H840) are shown in this figure.

**Figure S3**. Diverse genomic contexts involving *cas9* genes. (A) The *cas9* gene is inserted in the subtype III-B CRISPR-Cas system. (B) A *cas9* gene coexists with the subtype I-F CRISPR-Cas system.


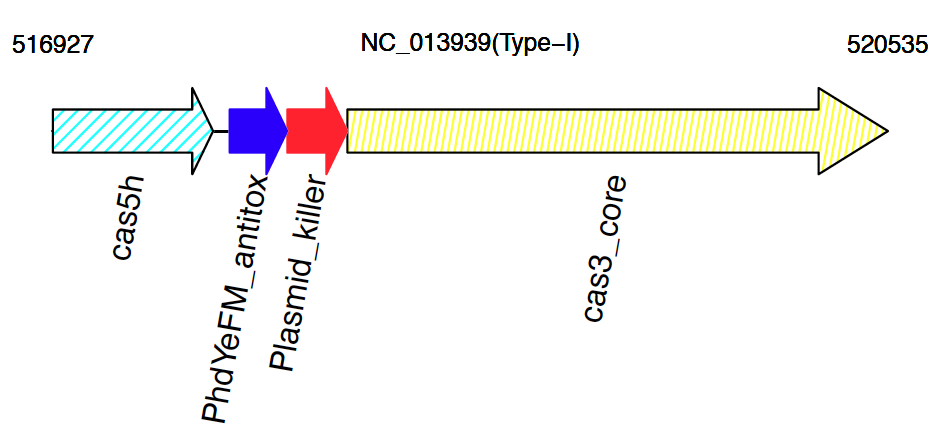

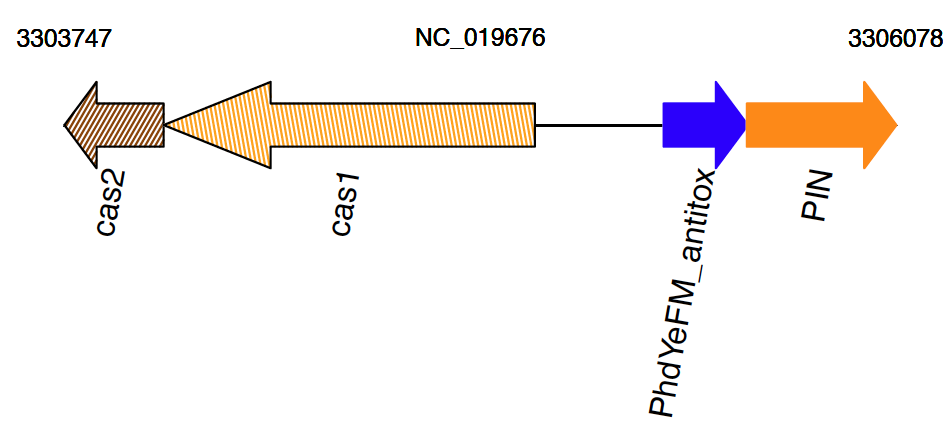

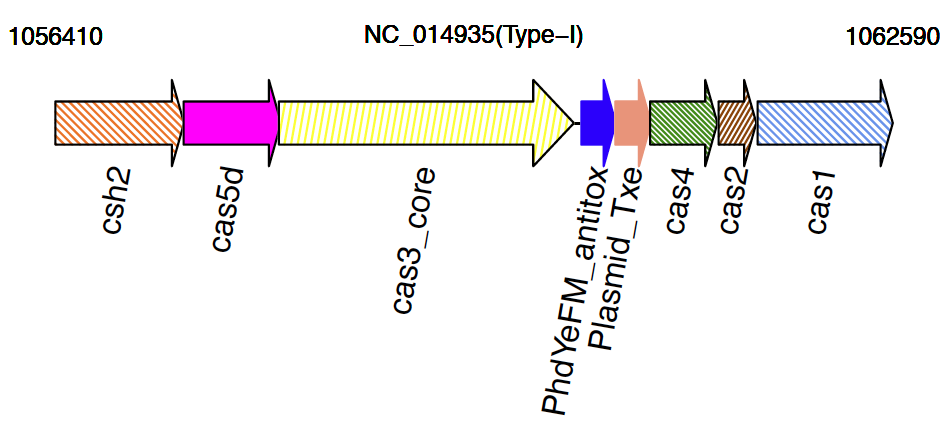

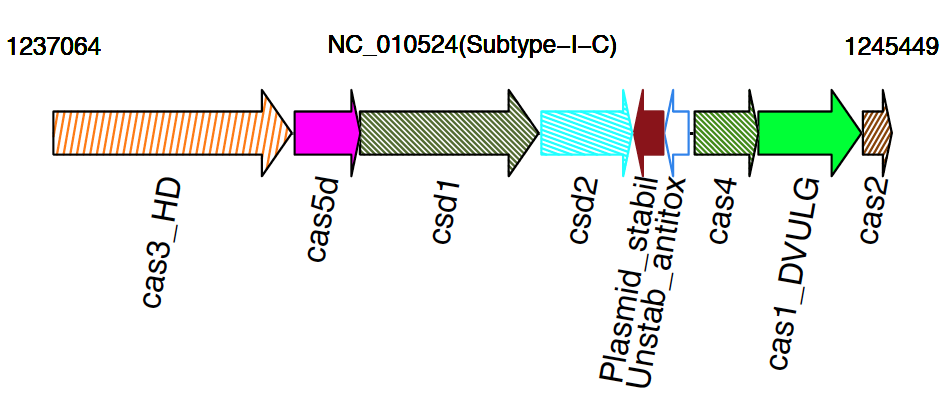

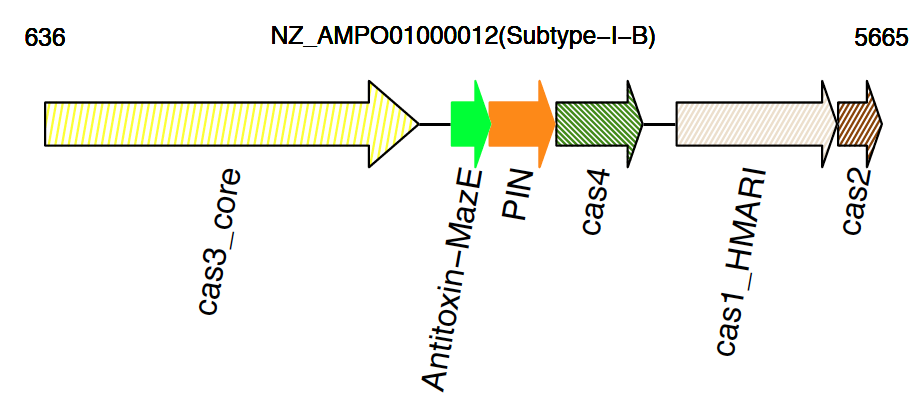

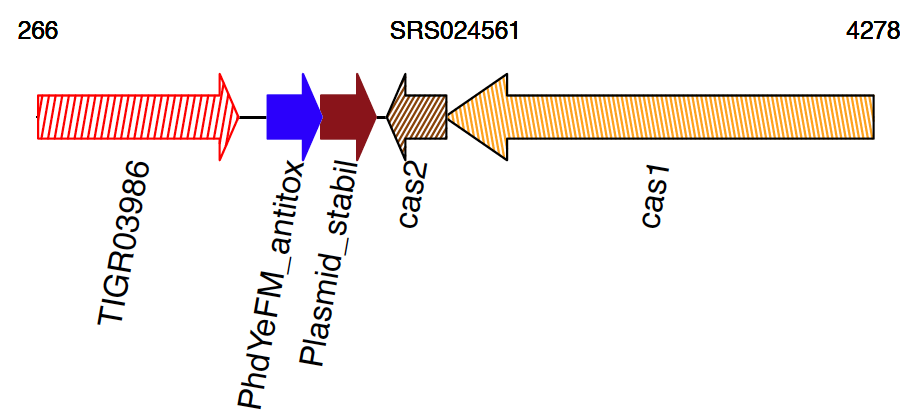

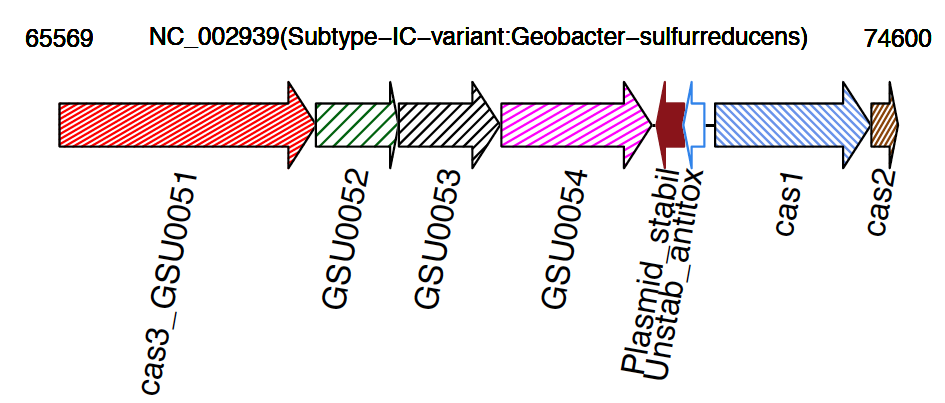


**Figure S4**. Examples of the toxin-antitoxin systems and their genomic contexts. Genes encoding antitoxins or toxins of the same family are shown in the same color. Genes are orientated such that known *cas* genes in the same locus are transcribed from left to right (thus, a toxin/antitoxin gene oriented from right to left is on the opposite strand of the *cas* genes). The subtypes were assigned based on subtype-signature genes.


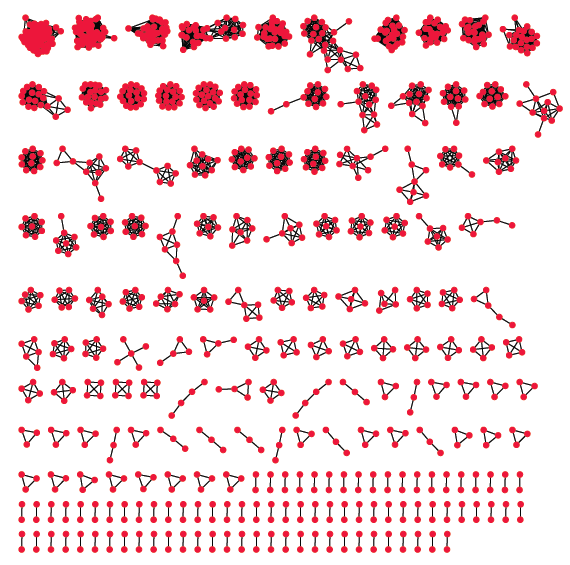


**Figure S5**. MCL clustering of all putative, unknown, Cas proteins, resulting in 208 clusters

**Figure S6**. Read coverage for selected *cas* genes identified from the HMP datasets.

**Figure S7**. The taxonomic assignment of all sequences collected from 24 novel families. The novel proteins were assigned to taxonomic groups by searching against the nr database. The tree was constructed using MEGAN 4 with BLASTP best hit, with a cut-off bit score of 60.

**A**

**B**

**
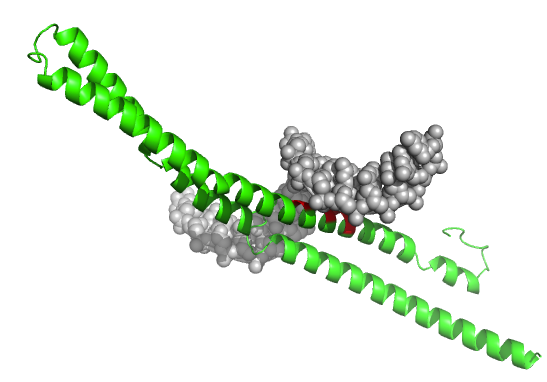
**

**Figure S8.** The conserved motif of one protein [the motif must be for a family](SRS022725.184166-T1-C) from Cash3 with phenylalanine and tyrosine rich (A). (B) The predicted tertiary structure of this protein is shown in green and four predicted DNA binding site residues were highlighted in red.
